# Serum Messenger RNA as a Biomarker and its Clinical Usefulness in Malignancies

**DOI:** 10.4137/cmo.s379

**Published:** 2008-09-22

**Authors:** Norimasa Miura, Junichi Hasegawa, Goshi Shiota

**Affiliations:** 1 Division of Pharmacotherapeutics, Department of Pathophysiological and Therapeutic Science, Faculty of Medicine, Tottori University, 86 Nishicho, Yonago, Tottori 683-8503, Japan; 2 Division of Molecular and Genetic Medicine, Department of Genetic Medicine and Regenerative Therapeutics, Graduate School of Medicine, Tottori University, Yonago 683-8503, Japan

**Keywords:** hTERT, real-time RT-PCR, cancer diagnosis, malignancy, tumor marker

## Abstract

A number of biomarkers are used clinically and many protein-based assay methods are available. Improvements in the method to utilize specific antibodies have led to remarkable progress in clinical diagnosis using biomarkers. Proteomics studies to identify better biomarkers have been performed worldwide by using a protein-based comprehensive method. The detection rate of conventional biomarkers can not improve further. Now is a time that a breakthrough is needed. We previously proposed mRNA, which is circulating in the body, as a novel material for biomarkers. mRNA is an unexpectedly useful molecule, not only because it can detect genes with a low expression level in protein, but also because it can detect the expression from non-coding RNA precursor genes or gene products with limited secretion from the cells. Circulating mRNA has been thought to be unstable in blood containing RNase. We confirm that mRNA remains at the same level for 24 hours after blood sampling. Unlike DNA, the RNA molecule can reflect events in the human body which occurred within a day, resulting in an early diagnosis of diseases. We report the possibility to detect and quantify cancer-derived mRNAs circulating in human vessels. We introduce the detection of serum mRNA as a useful biomarker of human malignancies.

## Background of Circulating Nucleic Acids

Since the discovery of circulating nucleic acids in plasma in 1948, many diagnostic applications have emerged. Small amounts of circulating nucleic acids (CNA) are present in the plasma of healthy individuals. Increased levels of plasma CNA have been reported in a number of clinical disorders such as cancer, stroke, trauma, myocardial infarction, autoimmune disorders and pregnancy-associated complications. CNA has received special attention because of its potential application as a non-invasive, rapid and sensitive tool for molecular diagnosis and monitoring of the diseases, and the prenatal diagnosis of fetal genetic diseases. A simple blood test for cancer detection has been the quest of many researchers in particular. Recently, CNA instead of a protein has been used in practical diagnosis. Cell-free circulating nucleic acids in plasma/serum derived from tumor tissues, have received much interest. Although it is well known that higher concentrations of DNA (deoxyribonucleic acid) are present in the plasma of cancer patients sharing some characteristics with DNA of tumor cells (1, 2, 3, 4), it has been reported that mRNAs detected in blood reflects the early event in a clinical condition (5). Since RNA (ribonucleic acid) in plasma/serum may be a suitable source for the development of non-invasive diagnostic, prognostic and follow-up tests for cancer, this discovery has provided us with very promising assays useful for early detection of malignancies.

## Circulating RNA as Diagnostic Source

A recent development in this new field is the identification of tumor-related RNA in the plasma/serum of cancer patients (6). These include tyrosine kinase mRNA (7), telomerase components (8, 9), the mRNAs that are encoded by different tumor-related genes (10, 11, 12, 13, 14), and viral mRNA (14). As more RNA than DNA markers can be detected in the circulation of cancer patients, an assay using RNA markers produced higher sensitivity than other conventional assays. In one study, two telomerase markers of breast cancer yielded 44% positive rates (8). However, telomerase RNA seems to be a promising marker as it can be detected even in the serum of patients with small, undifferentiated breast cancers without any metastatic lesions. Dasi et al. showed that circulating telomerase RNA is a sensitive marker, using real-time reverse transcription-polymerase chain reaction (real time RT-PCR) (9). In their study, 8 of 9 plasma samples from colorectal cancer patients and 9 of 9 plasma samples from patients with lymphoma tested positive for human telomerase reverse transcriptase. The plasma samples of all 10 healthy individuals were negative.

## Methods

**Figure 1:**
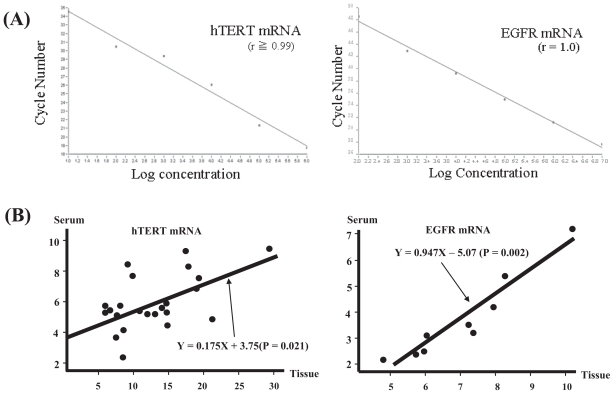
**(A)** In each quantitative assay, a strong linear relation was demonstrated between copy number and PCR cycles using RNA controls for concentration (r = 0.99 for hTERT mRNA: left; r = 1.0 for EGFR mRNA: right). The dynamic ranges of real-time PCR analysis for hTERT mRNA and EGFR mRNA were more than approximately 5~10 copies in this assay and we were able to exclude the possibility of false negativity in serum samples from patients and controls. Control hTERT mRNA for standardization was generated using T7 RNA polymerase in pLIXN-hTERT cDNA kindly provided from Dr. H. Tahara (Hiroshima University, Japan) and another control EGFR mRNA was similarly generated using pCRII-TOPO-EGFR (Invitrogen Japan K.K, Tokyo, Japan) retrofitted from pME18SFL3-EGFR purchased as FLJ cDNA clone commercially (TOYOBO, Tokyo, Japan). **(B)** A dot plot represents the significant correlation of (left) hTERT mRNA level in serum in lung cancer tissues in 23 patients and of (right) EGFR mRNA level in serum in lung cancer tissues in 9 patients. Only a minority of the cases that were positive for mRNA in the tissue specimens (n = 23 for hTERT, n = 9 for EGFR) is included in this analysis. Positive is defined as “above the predictive cut-off values for both mRNAs obtained from this study in 112 lung tumors and 80 healthy individuals”. These data were analyzed by the paired t test (p < 0.01 for both) and non-parametric Spearman’s test (p = 0.021 for hTERT mRNA, p = 0.002 for EGFR mRNA, respectively). The data were evaluated by logarithm of quantification.

To detect a transcript of interest in cell-free serum, quantitative real-time RT-PCR was performed by using 1 μl of RNA extract and 2 μl of SYBR Green I (Roche, Basel, Switzerland) in a One Step RT-PCR kit (Qiagen, Tokyo, Japan) using LightCycler with reproducibility. After blood sampling, RNA was extracted with DNase treatment after three steps of centrifugation of serum as previously reported with a few modifications mainly including a precise gravity control of centrifugation to obtain cell-free serum and mRNA quantification using optimized primer set (INTEC Web and Genome Bioinformatics, Tokyo, Japan) (8, 16). Other minor modifications are as follows. RNA from 200 μl of serum was dissolved in 200 μl of water. Quantitative RT-PCR was performed by using 1 μl of RNA extract and 2 μl of SYBR Green I (Roche, Basel, Switzerland) in a One Step RT-PCR kit (Qiagen, Tokyo, Japan). RNA in serum was extracted by using the same volume of serum and dried up to 20-fold concentration. RNAs from tissues were extracted using TRIzol Reagent according to manufacture’s instructions (Invitrogen Corp., Carlsbad, CA, U.S.A.). RT-PCR consisted of an initial incubation at 50 °C for 30 min followed by a 12-min incubation at 95 °C, then 50 cycles at 95 °C (0 s), 55 °C (10 s), and 72 °C (15 s), and a 20 second melting at 40 °C. The dynamic ranges of real-time PCR analysis for mRNA of interest were more than approximately 5 to 10 copies in this assay. We could therefore exclude the possibility of false negative results in serum samples from patients and controls. To examine significant clinicopathological findings affecting mRNA and other markers, the difference among diseases and stratified categories in each clinical parameter was statistically evaluated. Tumors, tumor size, number of tumors, histological findings including differentiation degree of tumors, clinical staging, and the presence of metastasis were analyzed. Correlations among biomarkers were calculated using Pearson’s relative test. To assess the accuracy of diagnostic tests, the matched data sets (patients with reference diseases or healthy individuals and those with malignancies) for bio-markers were analyzed using receiver operator characteristic (ROC) curve analysis. This assay demonstrated a strong linear relation between copy number and PCR cycles using RNA controls (r^2^ > 0.99) (Fig. 1A). A correlation of mRNA between malignant tissue and serum was analyzed to examine whether serum mRNA is derived from the original tissues by both paired t test and Spearman’s test. Optimal predictive cut-off values and the sensitivity/specificity for mRNA expressions, and positive predictive value (PPV)/ negative predictive value (NPV) during carcinogenesis were calculated. All the primers were optimally designed (INTEC Web and Genome Informatics corp., Tokyo, Japan).

## Biomarkers in Hepatocellular Carcinoma

### Introduction

HCC is a highly fatal cancer that affects approximately half a million people worldwide (17, 18). The incidence of HCC has been rising rapidly and this increase has generated concern among clinicians, researchers and policy makers. Major risk factors of HCC include hepatitis C virus (HCV), hepatitis B virus (HBV), nonalcoholic steatohepatitis (NASH) and heavy alcohol consumption. Development of HCC is generally thought to depend on the rapid and long-term repeated cell turnovers. This indicates that HCC expands clonally from the hepatocytes which have been suffering intense inflammation for a long duration, causing senescence. It would be advantageous to detect the evidence of clonal occurrence of HCC at an early stage using a newly developed modality.

### Tumor markers for HCC

AFP has been used as a serum marker for HCC in humans for many years and has a sensitivity of 39%–65% and specificity of 76%–94% (19). However, the detection rate can be improved using ultrasonography (US) (20). Several biomarkers such as DCP, AFP-L3 (21), human hepatocyte growth factor(HGF), and insulin-like growth factor-1(IGF-1) are promising, but none of these markers have been validated for clinical use compared to AFP. There is an urgent need for novel biomarkers for the detection of early-stage HCC (22). The marker should be specific for HCC, enabling the detection of HCC at an early stage, easily measured and the test should be reproducible, minimally invasive and acceptable to patients and physicians (23). Among biomarkers identified at this stage, DCP and AFP-L3 have been most extensively studied and seem to be promising. DCP has a sensitivity of 28% to 89% and specificity of 87% to 96%. AFP-L3 has a sensitivity of 36% to 96% and specificity of 89% to 94%. Most of these markers are measured by enzyme immunoassay (EIA) to detect proteins due to the advantage of its stability.

### Application of the hTERT mRNAs in blood for diagnosis

The telomerase enzyme complex has two major subunits and its expression is mainly regulated by a catalytic subunit (human telomerase reverse transcriptase, hTERT) (2). Since many kinds of mRNAs could be found in the plasma and serum of healthy individuals and cancer patients (3), it has been suggested that the detection of cancer-related gene expression in the serum is very useful for the diagnosis and follow-up of patients. The hTERT mRNA or endogenous RNA component derived from cancer cells did not seem to be detectable in serum due to its instability by RNase (ribonuclease) in serum. However, since RNAs in serum are stable at most for 24 hour after drawing blood (24, 25), it has been suggested that they can even be detected in blood by a narrow margin (7, 26). hTERT mRNA can be detected in serum from breast cancer patients and its maximum sensitivity and specificity were at most 40% and 100% respectively (8). We previously reported that approximately 88% was qualitatively positive for the detection of HCC-derived RNA in serum (16), indicating that serum RNA can be applied for the diagnosis of other cancers as well as HCC. We singled out hTERT mRNA as the most appropriate molecule for cancer diagnosis. In this context, we focused on HCC, since hTERT mRNA is gradually increased during the multi-step process of hepatocarcinogenesis (27).

### Materials, methods, and results of hTERT mRNA in HCC

**Figure 2:**
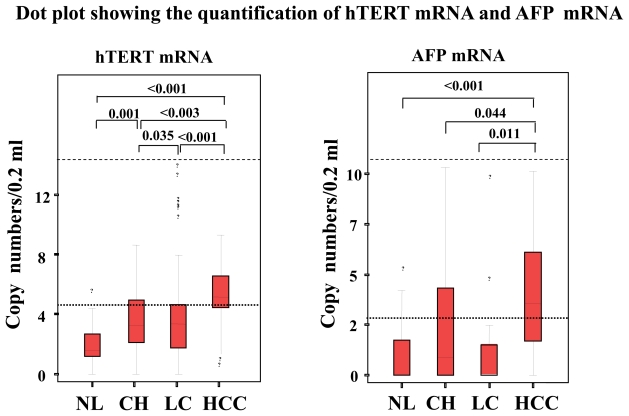
(left) hTERT mRNA levels and (right) AFP mRNA level (on logarithmic scales) in serum from patients with HCC, LC, CH, and healthy individuals by real-time RT-PCR. The 95% confidence interval in each group is shown beside the dots. Significant differences between 4 groups are shown in the upper part of the figure. NL, individual with normal liver: OL, other liver diseases: CH, chronic hepatitis: LC, liver cirrhosis: HCC, hepatocellular carcinoma.

**Figure 3:**
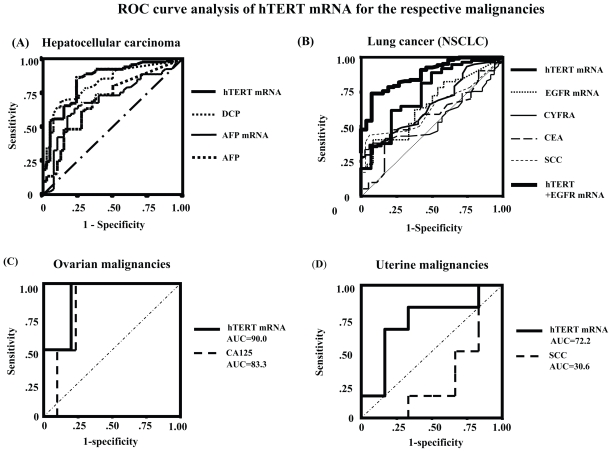
Receiver operator characteristic (ROC) curve analysis of the hTERT mRNA and/or EGFR mRNA expressions in comparison with conventional tumor markers. The curves shown were obtained by processing quantified raw data by SPSS II software and the sensitivity/ specificity values were predicted from the area under the curves and the calculated data. **(A)** For hepatocellular carcinoma, **(B)** for lung cancer (NSCLC), **(C)** for ovarian malignancies, and **(D)** for uterine malignancies.

This study enrolled 104 consecutive patients [64 patients with HCC, 20 with liver cirrhosis (LC), and 20 with chronic hepatitis (CH)]. All the HCC patients had LC as the underlying liver disease. The mean ages of patients with HCC, LC and CH were 66, 64 and 55 years respectively. Sixty-six patients were infected with HCV, 30 with HBV, 3 with both viruses and 5 with no viral markers. Patient gender, age, etiology, Pugh score, Child classification, underlying liver disease, total bilirubin (TB), albumin (Alb), alanine aminotransferase (ALT), AFP, AFP-L3, DCP, HCV titer, HCV subtype, tumor number, tumor size, differentiation degree of tumor, and presence of metastasis were evaluated. Fifty healthy individuals including 12 females (range 22–83 years old: mean age 58 years) served as controls. To study whether hTERT mRNA in serum is produced and released from HCC in the liver, we harvested the surgically resected HCC tissues and sera in another 10 HCC patients. Both hTERT mRNA and AFP mRNA expressions showed stepwise up-regulation with disease progression and the quantification was significantly higher in HCC than in LC, CH, and healthy individuals (p < 0.0001, p < 0.0001 and p < 0.0001 in hTERT; p = 0.011, p = 0.044 and p < 0.0001 in AFP, Fig. 2). Alb, tumor size, number of tumors, degree of differentiation of tumors and the presence of metastasis were significantly associated with hTERT mRNA expression (p < 0.05, p < 0.01, p < 0.0001, p < 0.0001 and p < 0.05, respectively, Table 1). To examine correlations among biomarkers, we calculated Pearson’s relative test and found that hTERT mRNA level was significantly correlated with AFP mRNA level (*P* < 0.05). Significant correlation of hTERT mRNA in HCC tissues with that in serum was also observed (p < 0.01, Fig. 1B). DCP (p < 0.05), AFP level (p < 0.05) and AFP-L3 (p < 0.05) showed a significant correlation with tumor size when it was stratified as <20 mm, 20–30 mm and >30 mm in diameter. Furthermore, hTERT mRNA expression was closely associated with well- and moderate-degree of differentiation of HCC (p < 0.05). hTERT mRNA were superior to other tumor markers in differentiating HCC from chronic liver disease by Friedman’s test (p < 0.01). ROC curve analyses showed that the sensitivity/specificity of hTERT mRNA for HCC were 88.2%/68.7% (Fig. 3A). At that time, optimal predictive cut-off values for both mRNA expressions were 12 500 copies/0.2 ml and 3000 copies/0.2 ml, respectively. In the assay, the sensitivity/specificity in each tumor marker during hepatocarcinogenesis is shown in Table 2B. Positive predictive value (PPV)/negative predictive value (NPV) during hepatocarcinogenesis was 0.862/0.870 in hTERT mRNA. PPV/NPV in AFP mRNA, AFP level, AFP-L3 and DCP is 0.695/0.741, 0.812/0.389, 0.778/0.277 and 0.852/0.405 respectively. Control hTERT mRNA for standardization was generated using T7 RNA polymerase in pLIXN-hTERT cDNA.

#### Summary

Currently available tumor markers for hepatocellular carcinoma (HCC) are α-fetoprotein (AFP), lens culinaris agglutinin-reactive AFP (AFP-L3), and des-γ-carboxy prothrombin (DCP). However, the diagnostic potential of these markers cannot surpass abdominal ultrasonography (US) as modalities to detect small HCC in the early stage. There is a need to develop additional sensitive markers to improve the early detection of HCC. We introduce a newly developed quantitative method for detecting serum hTERT mRNA, which has a clinical significance in HCC diagnosis.

In 154 subjects, including 64 with HCC, 20 with liver cirrhosis, 20 with chronic hepatitis, and 50 healthy individuals, we measured serum hTERT mRNA using the newly developed real-time quantitative RT-PCR with SYBR Green I. Briefly, we examined its sensitivity and specificity in HCC diagnosis, clinical significance in comparison with other conventional tumor markers than mRNAs, and its correlations with the clinical parameters by using multivariate analyses and Friedman’s test.

Serum hTERT mRNA showed higher values in patients with HCC than in those with chronic liver diseases. hTERT mRNA expression was demonstrated to be independently correlated with clinical parameters such as tumor size (p < 0.001), number (p < 0.001) and differentiation degree (p < 0.001). The sensitivity and specificity of hTERT mRNA in HCC detection were 88.2% and 70.0% respectively. hTERT mRNA proved to be superior to AFP mRNA (71.6 and 67.5), AFP (69.3 and 60.0) and DCP (81.5 and 63.5), respectively (Table 2-A). Importantly, hTERT mRNA in serum was correlated with that in HCC tissue.

Serum hTERT mRNA is a novel and available marker for HCC detection. We are conducting a large-scale study with approximately 500 patients on the feasibility of HCC diagnosis. This method will be suitable for a number of hTERT-positive malignancies, and for the quantification of the expression of genes of which protein products are weakly expressed.

## Biomarkers in lung cancer (non small cell lung cancer: NSCLC)

### Introduction

Lung cancer is the leading cause of malignancy-related mortalities (28) with little change in the survival rates over the past two decades (29). Non-small cell lung cancer (NSCLC) now accounts for about three-quarters of all cases of lung cancer (30). Most patients die of progressive metastatic disease despite the development of new therapeutic strategies and advances in surgical treatment. Serum tumor markers are non-invasive diagnostic tools for malignant tumors and they are commonly used for the screening of cancer and as an indicator of the treatment-effect. In small cell lung cancer (SCLC), neuron-specific enolase (NSE), and progastrin-releasing peptide (proGRP) are effective markers. In NSCLC, carcinoembryonic antigen (CEA), squamous cell carcinoma related antigen (SCC) and cytokeratin 19 fragment (CYFRA 21-1) are commonly used for screening. At least one marker among CEA, SCC and CYFRA is positive in approximately 70% of patients with NSCLC (30). According to the histological category, the positive rates of CEA and CYFRA are high in adenocarcinoma (ADC) patients, and the positive rates of CYFRA and SCC are high in squamous cell carcinoma patients. Although the standard diagnostic procedures such as X-ray examinations, conventional tumor markers and bronchial lavage (BL) are important for the detection of lung cancer, they are still not sensitive enough to detect lung cancer at an early clinical stage.

Tyrosine kinase activity of epidermal growth factor (EGFR) promotes tumor cell proliferation, cell survival, angiogenesis, invasion, and metastasis, and its specific inhibition by gefitinib, a synthetic anilinoquinazoline, has been demonstrated (32). EGFR is expressed in 20% to 80% of tissue specimens of NSCLC (33), but its expression has been observed in progressive type of SCLC (34). EGFR may therefore be a potential molecule for diagnosis or a good target that determines responsiveness to EGFR-targeted therapies (35). Some groups have reported that cell-free circulating hTERT mRNA in plasma can be detected in 12% of patients with lung cancer (NSCLC) (5), suggesting that hTERT mRNA detected in blood may be applicable for lung cancer as a diagnostic vehicle.

This study on pulmonary malignancy also demonstrates the clinical usefulness of hTERT mRNA, especially when combined with EGFR mRNA as a novel tumor marker in primary lung cancer for early detection and diagnosis. This method would be useful for patients or ethnic groups in which drugs targeting EGFR in lung cancer are thought to be effective.

#### Patients and sample collection

This study enrolled 89 consecutive patients with lung tumor (75/89 with NSCLC, 6/89 with SCLC and LCLC, and 8 with benign tumor) who were admitted to the National Hospital Organization Yonago Medical Center between July 2003 and December 2004 (Table 3). The mean age of the patients was 63 years (range 22 to 90 years). The patients were diagnosed based on serological examinations, chest X-rays, (helical) computed tomography (CT), chest and brain magnetic resonance imaging (MRI), cytological examinations, and transbronchial, percutaneous, and thoracoscopic lung biopsies. The final diagnosis was made using surgically resected specimens for pathology. Patient demographics, diagnostic tumor size, number of tumors, tumor markers including CEA, SCC, CYFRA, proGRP, NSE, TPA, SLX, history of smoking (estimated by Pack-Year index), the presence of metastasis or recurrence, and clinical stage (IA~IV) were evaluated. Twenty-seven healthy individuals including 12 females (range 22 to 90 years old: mean age 58 years) served as controls. To examine any change in gene expression in serum before and one month after surgical treatment in the same patients and to examine their significance as tumor markers, we quantified hTERT mRNA and EGFR mRNA expression in 9 patients with lung cancer. Control EGFR mRNA was generated using pCRII-TOPO-EGFR (Invitrogen Japan K.K, Tokyo, Japan) retrofitted from pME18SFL3-EGFR and purchased as a FLJ cDNA clone (TOYOBO, Tokyo, Japan).

### mRNA quantification and clinical parameters

In each quantitative assay, a strong linear relation was demonstrated between copy number and PCR cycles using RNA controls for concentration (r^2^ ≥ 0.99 for hTERT mRNA and EGFR mRNA; Fig. 1A). The copy numbers of hTERT mRNA (p < 0.01) and EGFR mRNA (p < 0.01) were significantly higher in the lung cancer patients than in the healthy individuals. Pearson’s relative test to clinical parameters denoted that the hTERT mRNA level was significantly associated with the presence of active liver disease, TPA, SCC and number of tumors (p < 0.05, p < 0.05, p < 0.001, and p < 0.05, respectively). EGFR mRNA was significantly correlated with the number of occupied lobular segment, metastasis, recurrence, and clinical staging (p < 0.05, p < 0.05, p < 0.01, and p < 0.01, respectively). A comparison of hTERT-negative (less than the predictive cut-off value) patients with hTERT-positive (more than the predictive cut-off value) patients, hTERT-negative patients were significantly correlated only with a pathological diagnosis (ADC) in the other clinical factors by Kruskal-Wallis test (p = 0.02, respectively).

### Statistical analysis and ROC curve analysis

Clinicopathological findings that were analyzed by one-way ANOVA showed significant relations to hTERT and EGFR expression in the serum (Table 3). Smoking, tumor size, number of tumors and the presence or absence of metastasis or recurrence, was significantly associated with hTERT mRNA expression (p < 0.05, p < 0.01, p < 0.01, p < 0.01 and P < 0.05, respectively). The number of tumors (p < 0.05), tumor size (p < 0.05), recurrence (p < 0.05) and clinical staging (p < 0.05) were significantly correlated with EGFR mRNA expression. In lung cancer, all tumor markers showed no significant relations with pathological diagnosis. CEA level showed a significant correlation with smoking (p < 0.05) (36). SCC level was correlated with the number of tumors (p < 0.05), tumor size (p < 0.05) and metastasis (p < 0.05). CYFRA level was correlated with the number of tumors (p < 0.05), tumor size (p < 0.05), metastasis (p < 0.05) and recurrence (p < 0.05). To examine the sensitivity and specificity of tumor markers for diagnosis of lung cancer, an ROC curve analysis was performed (Fig. 3B). The sensitivity of CEA, SCC, CYFRA, EGFR mRNA and hTERT mRNA for lung cancer was 40.1%, 58.9%, 48.8%, 60.8% and 71.8%, respectively (Table 4). On the other hand, the specificity of CEA, SCC, CYFRA, EGFR mRNA and hTERT mRNA was 74.4%, 58.3%, 74.2%, 62.5% and 72.5%, respectively. The positive predictive value (PPV) and negative predictive value (NPV) of hTERT mRNA were the highest of the tumor markers examined. Furthermore, the combination of hTERT mRNA and EGFR mRNA improved the sensitivity and specificity (PPV and NPV) to 82.8%, 77.7%, 89.8%, and 73.7%, respectively. The optimal cutoff values for hTERT mRNA and EGFR mRNA were calculated as 10^3.76^ copies/0.2 ml and 10^2.81^ copies/0.2 ml, respectively. The cut–off values of hTERT mRNA combined with EGFR mRNA, which did not significantly correlate with each other, were calculated as the summation of both measurements prior to the logarithm of quantification, and showed no relative significance to each other. The combination of hTERT and EGFR mRNA was superior to any of the other tested serum markers used for isolation.

### Correlation of hTERT mRNA and EGFR mRNA detection in paired serum and tumor tissue samples

The copy number of hTERT mRNA in serum was significantly correlated with that in cancer tissue (p < 0.05). The copy number of EGFR mRNA in the serum was significantly related to that in the cancer tissue specimens (p < 0.01), Fig. 1B). The data suggest that both RNAs in the serum are derived from lung cancer tissue specimens.

### Evaluation of serum hTERT quantification as a tumor biomarker

**Figure 4:**
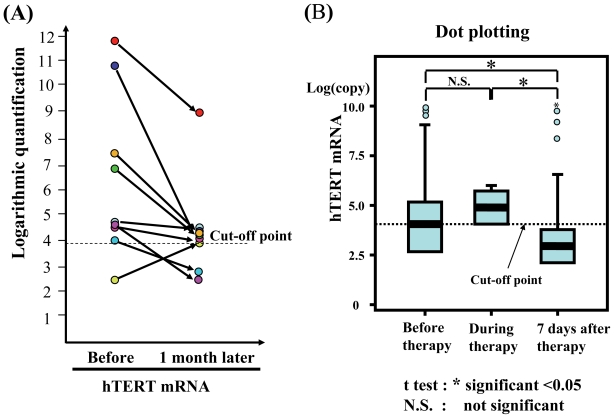
**(A)** A dot plot representing the significant correlation between hTERT mRNA level in serum and that in gynecologic cancer tissues in 9 patients. Only a minority of the cases that were positive for mRNA in the tissue specimens (n = 8 for hTERT) is included in this analysis. Positive is defined as “being above the predictive cut-off values for both mRNAs obtained from this study in 89 lung tumors and 27 healthy individuals”. These data for hTERT mRNA were analyzed by Wilcoxon’s test and the paired *t* test (p < 0.028 and p = 0.035, respectively). The data are evaluated by a logarithm of quantification. **(B)** The quantification of both mRNAs in the serum before, during, and 7 days after any treatment including chemotherapy or surgical treatment is stratified into three groups. The data are evaluated by a logarithm of quantification. hTERT mRNA expression among the three groups was evaluated by the paired *t* test (*p < 0.05). N.S. means not significant.

The quantitative decrease in hTERT mRNA one month after surgical treatment, compared with that before the treatment shown in Fig. 4A, suggests that hTERT mRNA is a useful biomarker which can be applied to cancer patients. Although EGFR mRNA tended to decrease after the treatment, this decrease was not statistically significant.

#### Summary

We attempted to clarify its clinical significance as a biomarker for lung cancer. In 89 patients with lung cancer and 27 individuals without, we measured serum hTERT mRNA and epidermal growth factor receptor (EGFR) mRNA levels, using a quantitative one-step real-time RT-PCR assay. We examined its sensitivity and specificity in lung cancer diagnosis, its clinical significance in comparison with other tumor markers, and its correlation with the clinical parameters using multivariate analyses and the correlation relative test. The copy number of serum hTERT mRNA was independently correlated with tumor size (p < 0.05), tumor number (p < 0.05), the presence of metastasis and recurrence (p < 0.05) and smoking (p < 0.05). EGFR mRNA correlated with tumor size (p < 0.05), tumor number (p < 0.05), recurrence (p < 0.05) and clinical stage (p<0.05). The sensitivity/specificity in lung cancer diagnosis was 71.8%/72.5% for hTERT mRNA and 60.8%/62.5% for EGFR mRNA respectively. hTERT mRNA was superior to other tumor markers in lung cancer diagnosis. Both mRNAs in serum were significantly correlated with those in lung cancer tissues (p < 0.05 for hTERT; p < 0.05 for EGFR). The copy number of hTERT mRNA significantly decreased after the surgical treatment. The combination of both mRNAs improved the sensitivity/specificity to 82.8%/77.7%, suggesting that hTERT mRNA, especially when combined with EGFR mRNA, is a novel and excellent biomarker for pulmonary malignancies to diagnose and assess the clinical stage and effects of treatments.

## Biomarkers in Gynecologic Malignancies

### Introduction

Ovarian cancer is the fifth most common cancer in women. Despite the fact that it is highly curable if diagnosed early, ovarian cancer kills more women each year than all other gynecologic malignancies (37). There are no proven methods of prevention, and it is often a rapidly progressive and fatal disease. The only validated marker for ovarian cancer is CA125, which is detectable in the serum of more than 80% of women with ovarian cancer. In contrast, cervical cancer is the third leading cause of cancer death in women. Over half a million new cases are diagnosed every year worldwide. The most common histological type of cervical cancer is squamous cell carcinoma which accounts for more than 80% of all cervical cancers. An increasing number of reports indicate that other factors are involved along with human papilloma virus (HPV) to induce cervical carcinogenesis. A routinely used biomarker for advanced cervical cancer is SCC, which is detectable in the serum of less than 50% of women with cervical cancer. CA125 and SCC are reliable only in monitoring the response to treatment or recurrence, but not as a diagnostic or prognostic marker (38). Thus, there is considerable interest in identifying molecular diagnostic and prognostic indicators to guide treatment decisions.

It is well known that one of the carcinogenic biomarkers, human telomerase reverse transcriptase (hTERT) (39, 40), is not only expressed in mild dysplastic lesions in cervices (41), but is also often expressed in gynecological malignancies.

### Patients and sample collection

A total of 176 consecutive patients (47 patients with ovarian cancer, 63 with uterine cancer, 2 with other gynecologic cancer, 2 with border lesions, and 62 with benign diseases) that were admitted to Tottori University Hospital between December 2003 and January 2005, were enrolled in this study. Of the patients with benign disease, 41 had benign ovarian diseases including 11 with an ovarian dermoid cyst, 4 had endometriosis in the ovary, 13 had an ovarian cyst and 21 had benign uterine diseases including 1 patient with invasive hydatidiform mole, 4 with adenomyosis of the uterus, 14 with uterine myoma and 2 with uterine prolapse. Twenty healthy patients served as controls. The mean age of the patients was 55 years (range 18 to 85 years). CA125 (ChemilumiACS-CA125II, Bayer Japan, Tokyo) and SCC (SCC RIAbeads, SRL, Tokyo, Japan) in the serum were measured in routine laboratory tests. Human papilloma virus (HPV) was not examined for uterine lesions. The patients were diagnosed by chief complaints, ultra-sonography, computed tomography (CT), CA125 or SCC, cytology or biopsy under internal examination, histological examination after laparoscopy or surgical therapy. The clinicopathological findings, gender, age, etiology, histological findings, CA125 for ovarian disease, SCC for uterine disease, tumor size, clinical staging, and presence of recurrence were evaluated.

### mRNA quantification and clinical parameters

According to this quantitative assay, the copy numbers of hTERT mRNA were significantly higher in the gynecologic cancer patients than in the healthy individuals (p < 0.01, each). Clinicopathological findings showed significant relations to hTERT mRNA in the serum (Table 5). By multivariate analysis, the hTERT mRNA level was significantly correlated with age, the presence of cancer, ovarian disease in organs, ovarian malignancies, tumor size, and CA125 (p < 0.001, p = 0.004, p = 0.045, p = 0.004, p = 0.044, p = 0.035 and p < 0.001, respectively). On the other hand, EGFR mRNA did not show any significant correlation with any parameters or with other markers. SCC was significantly associated with uterine malignancies (p = 0.021). With the Friedrich test, hTERT mRNA, SCC, and CA125 were significantly correlated with the clinical stage (p < 0.001, p = 0.033, and p = 0.028, respectively). Pearson’s relative test between clinical parameters revealed that the hTERT mRNA level was significantly associated with the presence of cancer (p = 0.009) and, in the ovary, with CA125 and age (p = 0.049 and p = 0.045, respectively).

To examine the sensitivity and specificity of tumor markers for diagnosis of gynecologic malignancies, ROC curve analysis showed the sensitivity/specificity of hTERT mRNA for gynecologic malignancies to be 74.4%/74.1% (data not shown). For ovarian malignancies, area under the curve (AUC) of hTERT mRNA and CA125 were 90.9% and 83.3%, respectively, and the sensitivity/specificity of hTERT mRNA in ovarian cancer is 95.0%/90.0% (Fig. 3C). The sensitivity/specificity of hTERT mRNA and SCC in uterine cancer is 70.1%/81.5% and 50.0%/67.5% respectively. On the other hand, the sensitivity/specificity of hTERT mRNA and CA125 in ovarian cancer is 100%/76.5% and 100%/75.5% respectively. For uterine malignancies, AUC of hTERT mRNA and SCC were 72.2% and 30.6% (Fig. 3D), respectively. The optimal cut-off values for hTERT mRNA and EGFR mRNA were calculated as 10^4.1^ copies/0.2ml and 10^2.99^ copies/0.2 ml, respectively.

### Evaluation of serum hTERT quantification as a tumor biomarker

All patients were stratified into three categories based on the timing of blood sampling (before, during, and after therapy) and the therapeutic effect such as anti-tumor agents or surgical treatment was estimated by a *t* test. Although there was no correlation between before and during blood sampling, hTERT mRNA significantly decreased after therapy, compared with before and during the therapy (p < 0.05, each) (Fig. 4B). This suggests that the measurement of hTERT may be useful for the evaluation of a therapeutic effect. In uterine malignancies, hTERT mRNA and SCC were significantly useful biomarkers to evaluate therapeutic effect (p = 0.001 and p = 0.026, respectively). In ovarian malignancies, hTERT mRNA and CA125 were significantly useful for the diagnosis of cancer (p = 0.001 and p = 0.043, respectively). The copy number of hTERT mRNA in serum was significantly correlated with that in cancer tissue (p = 0.028 in Wilcoxon’s test, p = 0.035 in the paired *t* test).

#### Summary

We attempted to elucidate the diagnostic evaluation of serum hTERT mRNA for gynecologic malignancies with our method. In 174 female patients with gynecological lesions (47 with ovarian lesions, 63 with uterine lesions, 2 with malignancies in other gynecological lesions, and 62 benign lesions) and 20 healthy individuals, we measured serum hTERT mRNA and EGFR mRNA by our real-time quantitative RT-PCR in the same way as lung cancer. We examined their sensitivity and specificity in cancer diagnosis, clinical significance in comparison with conventional tumor markers, and their correlations with the clinical parameters by using multivariate analyses. Serum hTERT mRNA showed higher values in patients with gynecologic cancers than in those with benign diseases and healthy individuals. The hTERT mRNA level independently correlated with the presence of cancers (p = 0.004 for both ovarian and uterine cancer) and clinical stage (p < 0.001). The sensitivity and specificity of hTERT mRNA in cancer diagnosis was 74.4% and 74.1%, respectively. The hTERT mRNA level showed a significant correlation with CA125 by Pearson’s relative test (p = 0.035) and with histological findings in ovarian cancer by the Friedrich test (p < 0.004). EGFR mRNA never displayed any differences between the diseases. hTERT mRNA is useful for diagnosing gynecologic cancer and is superior to conventional tumor markers. Therefore, serum hTERT mRNA is a novel and available biomarker for gynecologic malignancies.

## Discussion

In a subsequent quantitative study, we have improved the sensitivity to detect the instable nucleotides in blood by removing cellular proteins and minimizing the contamination of cellular nucleic acids in serum and a primer set which can amplify hTERT mRNA efficiently (35). Furthermore, the correlation between tumor tissue and serum in terms of hTERT mRNA was demonstrated in Figure 1B, suggesting that hTERT mRNA detected in serum is derived from tumor cells. AFP is widely used as a reliable marker of HCC, not in earlier stage but in advanced stage (42). Since HCC recurs repeatedly and polyclonally due to biological characteristics even after any treatments, the monitoring of serum hTERT mRNA might make it possible to diagnose the recurrence earlier. In this respect, we prospectively have to conduct a follow-up study after the treatment of HCC (manuscript in preparation). hTERT mRNA expression was found to be closely associated with a well to moderate degree of differentiation of HCC. Nakashio et al. previously reported a significant correlation between HCC differentiation and telomerase expression (43). The results in the present study confirmed their findings. hTERT mRNA showed more sensitivity and specificity compared with AFP mRNA in HCC diagnosis. AFP mRNA did more sensitivity and specificity compared with AFP level (5). The higher specificity of hTERT mRNA may be related to the fact that AFP mRNA is produced in HCC cells and injured hepatocytes. However, hTERT is produced mainly in HCC cells. Waguri et al proved that hTERT mRNA in circulating cancer cells, derived from HCC tissues, can be detectable by using a cell-sorting system that they developed. The authors indicated that HCC cells released from the original HCC were within 10 mm in size (44) and suggested that the value is consistent with the result of the mRNA detection method. Our studies suggest that quantification of hTERT mRNAs in serum has diagnostic implications for NSCLC and ovarian cancer as well as HCC. We will evaluate the correlation between prognosis and hTERT mRNA (45), and assess the availability of hTERT mRNA in other hypervascular cancers by comparing hTERT mRNA with other tumor markers. We are performing a large-scale study with more than 500 patients to confirm our results for the monitoring and detection of HCC. Since our assay is difficult to perform manually, we are developing an automatic, concise and rapid diagnostic apparatus with a reagents kit to be introduced into clinics, even at the primary care level. In the diagnostic field of medicine, current patent laws involved in intellectual property rights are limiting diagnostic progression. This method will be suitable for the diagnosis of a number of hTERT-positive malignancies and for cancer-specific RNA genes or other genes showing weak protein levels. The systemic introduction of this assay into the primary care level can be expected in the near future.

In pulmonary and gynecological malignancies, we suggest that the correlation with response/survival may be more evident if this more sensitive mRNA-based detection assay is used. The induction of a combination of hTERT mRNA and EGFR mRNA into the early diagnosis of lung cancer may improve the follow-up of patients. Since the data shows that hTERT and EGFR mRNA levels are associated with previously detected lung cancers, it does not demonstrate whether the method is sufficiently robust for early detection. However, the evaluation of hTERT mRNA combined with EGFR mRNA may be a useful biomarker to diagnose and assess the clinical stage and effect of treatments in patients with lung cancer. Further case-control studies should be planned to evaluate both long-term and former smokers who do not have lung cancer. hTERT mRNA is useful in the diagnosis of gynecologic cancers as well and is superior to conventional tumor markers. Therefore, serum hTERT mRNA is a novel and available biomarker for gynecologic malignancies.

## Investigations into other disease-specific mRNAs and the clinical significance

In other malignancies such as thyroid cancer and pancreatic cancer (in preparation), this assay has been applied (manuscript under preparation for publication). We are investigating other disease-specific mRNAs as biomarkers to be applied in the clinic or in primary care. The field of medical care we are engaged in is the diagnosis of intractable disease (Table 6). It is necessary to find improved mRNA such as hTERT, compared to Positron Emission Tomography (PET), as a diagnostic vehicle. We are studying inflammatory diseases such as fulminant hepatitis (46) or acute respiratory distress syndrome (in preparation), and lifestyle-related diseases, autoimmune diseases, and disease from gene dysfunction which are difficult to diagnose or estimate the clinical condition.

## Conclusion

Additional diagnostic methods (e.g. imaging), or combination of other biomarkers would be still needed to determine the type and location of the tumor. In our investigations, circulating mRNA is a very sensitive molecule to detect diseases in organs with rich blood flow or systemic inflammatory disease. The presence of acute phase diseases, including cancer progression, could be detected if mRNA is tested for within one day of blood sampling. However, this assay has a limitation. It can be influenced by organs with rich blood flow such as the liver. The disease of interest may be masked, for example in patients suffering from progressive liver disease. Based on these actual circumstances, it is likely that further developments over the next few years in the field of circulating RNA will provide us with new diagnostic and monitoring possibilities.

## Figures and Tables

**Table 1:** Statistical analysis of the comparison of hepatic tumor markers and clinicopathological findings.

Clinical parameters	# of patients	Multivariate analysis and Fredman test			
		hTERT mRNA	AFP mRNA	AFP@-L3@	DCP
		p	p	p	p
Age mean:59 years old (range 22 to 83)		0.408	0.798	0.681(0.690)	0.981
		0.761	0.089	0.412(0.408)	0.380
Gender					
M	94				
F	60	0.250	0.651	0.304(0.052)	0.842
Etiology					
HBV	30				
HCV	66				
HBV + HCV	3				
NBNC	2				
Alcohol	3	0.060	0.973	0.621(0.026)	0.534
Underlying lesion					
Normal	50				
CH	32				
LC	70	0.018	0.340	0.540(0.601)	0.001
Albumin (g/dl)		0.928	0.693	0.111(0.432)	0.933
Total bilirubin (mg/dl)		0.538	0.149	0.001(0.001)	0.978
Alanine aminotransferase (IU/l)		0.136	0.573	0.373(0.020)	0.001
Child-Pugh Scale					
A	21				
B	44				
C	5	0.201	0.319		
AFP (ng/ml)		0.123	0.425		
AFP-L3 (%)		0.854	0.651		
DCP (mAU/ml)		<0.001	0.061	0.358(0.001)	0.258
Size of tumor (mm)					
<20	18				
20~30	26				
> 30	20	<0.001	0.123	0.200(0.012)	0.086
Number of tumors					
1	10				
2	27				
3	27	0.010	0.096	0.011(0.010)	0.285
Degree of differentiation					
Well	33				
Moderate	27				
Undifferentiated	1				

Only hTERT mRNA correlated with albumin, tumor size, number of tumors, and degree of differentiation of tumor independently during the progress from chronic liver disease to HCC. HBV, hepatitis B virus: HCV, hepatitis C virus: NBNC, non -HBV non-HCV: AH, adenomatous hyperplasia.

**Table 2:** The sensitivity/specificity and PPV/NPV of each tumor marker for hepatocellular carcinoma are shown. PPV: positive predictive value, NPV: negative predictive value (**A**). Statistical significance in each tumor marker during hepatocarcinogenesis is shown (**B**).

A.				
	Sensitivity	Specificity	p value	PPV/NPV
AFP	0.693	0.600	0.002	0.812/0.389
AFP-L3	0.563	0.925	0.304	0.778/0.277
DCP	0.815	0.635	<0.001	0.852/0.405
AFP mRNA	0.716	0.675	<0.001	0.695/0.741
hTERT mRNA	0.882	0.700	<0.001	0.862/0.870

B.				
Tumor marker	CH/LC → HCC		LC → HCC	
	(n = 40)	(n = 64)	(n = 20)	(n = 64)
AFP level	0.376		0.532	
AFP-L3	0.144		0.228	
DCP	0.317		0.479	
AFP mRNA	0.001		0.001	
hTERT mRNA	<0.001		<0.001	

**Table 3:** Statistical analysis of the comparison between pulmonary tumor markers and clinical parameters.

Clinical parameters	No. of patients	One way ANOVA Bonferroni				
		hTERT mRNA	EGFR mRNA	CEA	SCC	CYFRA
		p	p	p	p	p
Age mean:63 years old (range 22 to 90)		NS	NS	NS	NS	NS
Gender						
M	72(15)	NS	NS	NS	NS	NS
F	44(12)					
Smoking						
Y	48	0.029	NS	0.031	NS	NS
N	41					
Number of tumors						
1	48	0.003	0.047	NS	0.016	0.017
2	13					
>3	26					
unknown	2					
Numbers of occupied segment		0.029	NS	0.031	NS	NS
1	50					
2	16					
>3	8					
unknown	15					
Diagnosis						
ADC	60	NS	NS	NS	NS	NS
SCC	15					
others	6					
(benign	8)					
Size of tumor						
<2 cm	22	0.002	0.043	NS	0.015	0.019
2~3 cm	28					
>3 cm	38					
unknown	1					
Metastasis						
Y	38	0.004	NS	NS	0.044	0.045
N	49					
unknown	2					
Recurrence						
Y	32	0.013	0.037	NS	NS	0.009
N	50					
unknown	7					
Staging (K-W test)						
I A, II B	12, 9	NS	0.032	NS	NS	NS
II A, II B	1, 7					
III A, III B	17, 1					
IV	5					

hTERT mRNA correlated with smoke, tumor size, number of tumors, metastasis, and recurrence, independently.

**Abbreviations:** K-W test, Kruskal-Wallis test: ADC, Adenocarcinoma: SCC, Squamous cell carcinoma related antigen: CEA, Carcinoembryonic antigen: CYFRA, Cytokeratin 21-1 fragment: NS, not significant. The numbers in parenthesis in the column of Gender indicate the number of healthy individuals.

**Table 4:** Sensitivity/specificity of each tumor marker for lung cancer.

	Sensitivity	Specificity	p value	PPV/NPV	Cut-off point
hTERT mRNA	71.8	72.5	0.006	77.5/66.7	3.76(10^x^ copy)
EGFR mRNA	60.8	62.5	0.023	66.7/40.7	2.81(10^x^ copy)
CYFRA	48.8	74.2	0.016	65.0/50.0	1.3(ng/ml)
SCC	58.9	58.3	0.032	20.7/87.5	1.5(ng/ml)
CEA	40.1	74.4	0.376	65.0/39.1	2.8(ng/ml)

hTERT + EGFR mRNA	82.8	77.7	0.001	89.8/73.7	5.38(10^x^ copy)

The sensitivity/specificity values are 71.8%/72.5% for hTERT mRNA, 60.8%/62.5% for EGFR mRNA, 48.8%/74.2% for CYFRA, 58.9%/58.3% for SCC, and 40.1%/74.4% for CEA. In the diagnostic assessment of sensitivity and specificity, hTERT mRNA (0.718/0.725) was identified as the most excellent tumor marker.PPV: positive predictive value, NPV: negative predictive value. Sensitivity, specificity, p value, and PPV/ NPV of hTERT + EGFR mRNA were calculated, based on the summation of each logarithmic cut-off values.

**Table 5:** Statistical analysis of the comparison between gynecologic tumor markers and clinical parameters.

Clinical parameters	# of patients	Multivariate analysis		
		hTERT mRNA	SCC	CA125
		p	p	p
Age mean: 55 years old (range 18 to 85 )		<0.001	N.S	0.028
Etiology				
malignant	89	0.004		
border	3			
benign	20			
Organ				
Uterus	52	N.S		
cervical	37			
body	5	0.045	N.S	N.S
Ovary	39			
Others	1			
Histological findings				
Uterus		N.S	0.021	N.S
Squamous cell carcinoma	29			
Endometrioid	15			
Others	22			
Ovary		0.004	N.S	<0.001
Serous	24			
Mucinous	9			
Others	22			
Tumor size		0.044	N.S	N.S
Tumor marker				
SCC(ng/ml)		N.S		N.S.
CA 125(mAU/ml)		0.035	N.S.	
Staging				
1	29	<0.001	0.033	0.028
2	8			
3	12			
4				
Before theraphy	60	N.S	N.S	0.043
During	6			
After	26			
Recurrence				
yes	33	N.S	N.S	N.S
no	59			

**Table 6:** Application of this assay for malignancies and other diseases.

**1. Application for malignancies**
a) A diagnosis of malignancies
Lung cancer
Adenocarcinoma
Squamous cell carcinoma
Small cell lung carcinoma
Large cell lung carcinoma
Gynecological malignancies
Ovarian cancer
Uterine cancer
Gastroenterological malignancies
Hepatocellular carcinoma
Stomach cancer
Colon cancer
Pancreaticcancer
Esophageal cancer
Breast cancer
Thyroid cancer
Otolaryngological cancer
Urinary cancer
Sarcoma
b) A Comparison of hTERT mRNA with PET in periodic medical examination for cancer
c) An evaluation of the induction or effect of anticancer therapy
**2. Application for other diseases**
a) Inflammatory diseases
Fulminant hepatitis
Autoimmune disease
Acute respiratory distress syndrome
Nonalcoholic steatohepatitis
Systemic inflammatory response syndrome
b) Ischemic diseases
c) Lifestyle-related diseases
Insulin resistance

## Abbreviations

hTERT: human telomerase reverse transcriptase protein
HCC: hepatocellular carcinoma
hTR: human telomerase RNA template
HCV: hepatitis C virus
HBV: hepatitis B virus
AH: adenomatous hyperplasia
AAH: atypical adenomatous hyperplasia
LC: liver cirrhosis
CH: chronic hepatitis
AFP: α-fetoprotein
DCP: des-γ-carboxy prothrombin
ALT: alanine aminotransferase
Alb: albumin
EGFR: Epidermal growth factor receptor
NSCLC: non-small cell lung cancer
SCLC: small cell lung cancer
ADC: adenocarcinoma
SCC: squamous cell carcinoma antigen
SqCC: squamous cell carcinoma
CEA: carcinoembryonic antigen
CYFRA (21–1): cytokeratin 19 fragment
CNA: circulating nucleic acids
